# Complete Genome Sequences of New Emerging Newcastle Disease Virus Strains Isolated from China

**DOI:** 10.1128/genomeA.00129-12

**Published:** 2013-02-21

**Authors:** Hualei Liu, Yan Lv, Claudio L. Afonso, Shengqiang Ge, Dongxia Zheng, Yunling Zhao, Zhiliang Wang

**Affiliations:** a OIE Reference Laboratory for Newcastle Disease, China Animal Health and Epidemiology Center, Qingdao, China; b Southeast Poultry Research Laboratory, United States Department of Agriculture, Athens, Georgia, USA

## Abstract

Five Newcastle disease virus strains isolated from geese were classified into a new genotype, designated genotype XII. The complete genome sequences of two strains indicated that these viruses were distinct from viruses of genotype VII. More investigations need to be conducted for us to understand the origin of these new strains.

## GENOME ANNOUNCEMENT

Newcastle disease (ND), caused by the virulent Newcastle disease virus (NDV), is a highly contagious disease that can cause significant losses in poultry worldwide. NDV is a member of the genus *Avulavirus* of the family *Paramyxoviridae* (1). The viruses comprise a diverse group with a single-stranded, negative-sense RNA genome. The genomic size of NDV has been predicted to be 15,186, 15,192, or 15,198 nucleotides (nt) in length (2, 3). NDV strains can be divided into two distinct clusters based on phylogenetic analysis of the fusion gene (4, 5). Class I and class II viruses have been recently classified into 1 and 15 genotypes, respectively (6).

NDV has caused four major ND panzootics since the first recognition of the disease in 1926. Different genotypes were involved in each panzootic. Genotype VII NDV strains represent the predominant genotype involved during the fourth panzootic since the late 1980s (7, 8). These strains also caused many outbreaks of ND in domestic geese with high mortality and morbidity in China since 1997, which has countered the traditional opinion that waterfowl are resistant to virulent NDV and only play the role of reservoirs (9–11).

During the national surveillance program of ND in 2010 and 2011, five NDV isolates were obtained from geese in the live-bird markets. The complete sequences of the F and HN genes of five isolates were determined by reverse transcription (RT)-PCR and direct sequencing. The amino acid sequence identities of the F and HN proteins among these five isolates ranged from 99.1 to 100% and 99.1% to 99.6%, respectively. The predicted amino acid sequences surrounding the cleavage site of F protein in all 5 isolates displayed the motif ^112^RRQKR↓F^117^, which is typical of virulent NDV isolates, and is in agreement with the results of *in vivo* pathogenicity tests. All 5 isolates had intracerebral pathogenicity index (ICPI) values of 1.74 to 1.93 in 1-day-old chickens.

Phylogenetic analysis based on the complete nucleotide sequences of the F and HN genes classified these isolates into a new genotype designated genotype XII (6), together with a strain isolated from Peru in 2008 (poultry/Peru/1918-03/2008), and showed that these viruses were distinct from viruses of genotype VII, the predominant genotype responsible for most outbreaks of ND worldwide during recent years.

The complete genome sequences were determined for two of these new emerging strains, namely Goose/GD1003/2010 and Goose/GD450/2011. The genomes of both strains are 15,192 nucleotides in length. Comparison of genome sequences showed that the highest genetic identity (91.3%) was observed with poultry/Peru/1918-03/2008 (GenBank accession number JN800306). Derived amino acid sequences of all proteins had 92.1% to 97.2% identity to the strain poultry/Peru/1918-03/2008, with the exception of P protein, for which the highest identity was only 85.3%. Although these new emerging strains belonged to the same genotype as the Peru strain, the large differences in the P protein gene, and the lack of an epidemiological relation between them, suggest that the origin of these emerging NDV strains is still not clear and more investigation must be conducted (12).

### Nucleotide sequence accession numbers.

The following genome sequences have been deposited in GenBank: Goose/GD1003/2010 (KC152049) and Goose/GD450/2011 (KC152048).
